# Supplementary material for: Causal Relationship between Aging and Anorexia Nervosa: A White-Matter-Microstructure-Mediated Mendelian Randomization Analysis
Source: Biomedicines. 2024 Aug 16;12(8):1874. doi: 10.3390/biomedicines12081874 (PMC11351342; doi:10.3390/biomedicines12081874)
Supplement: Supplementary file 1 [file biomedicines-12-01874-s001.zip › Supplementary Figure/Figure S8/Leaveoneout plot of MR analysis for TL to ubm-b-2079.pdf]

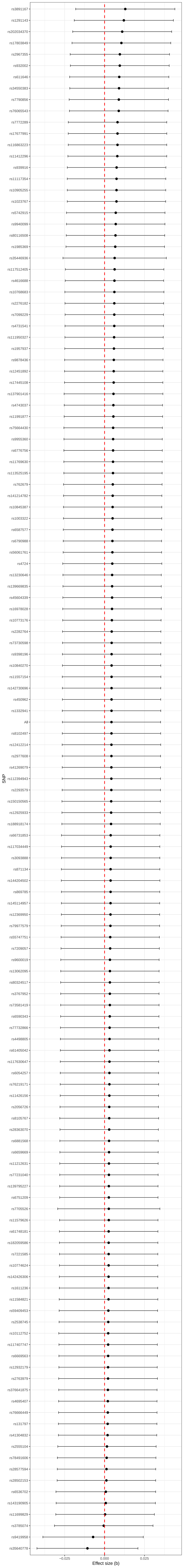

rs3891167
rs1291143
rs202034370
rs17803849
rs2967355
rs932002
rs611646
rs34550383
rs7790856
rs76065543
rs7772289
rs17677991
rs116863223
rs11412296
rs939916
rs11117354
rs10905255
rs1023767
rs5742915
rs9940099
rs80116508
rs1985369
rs35446936
rs117512405
rs4616688
rs10768683
rs2276182
rs7099229
rs4731541
rs111950327
rs1957937
rs9878436
rs12451892
rs17445108
rs137901416
rs4743037
rs11991877
rs75664430
rs9955360
rs6776756
rs11769630
rs113525195
rs762679
rs141214782
rs10845387
rs1003322
rs6587577
rs6790988
rs56061761
rs4724
rs13230646
rs139669835
rs45604339
rs16978028
rs10773176
rs2282764
rs73730598
rs9398196
rs10840270
rs11557154
rs142730696
rs450962
rs1332941
All
rs8102497
rs12412214
rs2977608
rs41269079
rs112394943
rs2293579
rs150150565
rs12925933
rs188918174
rs66731853
rs117034449
rs3093888
rs871134
rs144204502
rs869785
rs145114957
rs12369950
rs79977579
rs55747751
rs7209057
rs9600019
rs13062095
rs80324517
rs3767952
rs73581419
rs6590343
rs77732866
rs4498805
rs61405042
rs117630647
rs6054257
rs76219171
rs11426156
rs2056726
rs8105767
rs28363070
rs6881568
rs6659669
rs11212631
rs77231040
rs139795227
rs6751209
rs7705526
rs11579626
rs61748181
rs182059586
rs7221585
rs10774624
rs142426306
rs1611236
rs11584821
rs59409453
rs2538745
rs10112752
rs117407747
rs6669563
rs12932178
rs2763979
rs376641875
rs4695407
rs76666449
rs131797
rs41304832
rs2555104
rs78491606
rs28577594
rs28502153
rs6536702
rs143180905
rs11699829
rs3785074
rs9419958
rs35640778
SNP
-0.025
0.000
0.025
Effect size (b)
